# Burden of septic shock in the uk

**DOI:** 10.1186/2197-425X-3-S1-A154

**Published:** 2015-10-01

**Authors:** F Andersson, T Kristensen, AL Kjølbye

**Affiliations:** Ferring Pharmaceuticals A/S, Global Health Economics & Outcomes Research, Copenhagen, Denmark; Central Denmark Region, CFK, Aarhus, Denmark; Ferring Pharmaceuticals A/S, Clinical Research, Copenhagen, Denmark

## Introduction

Septic shock is a major health care problem which affects between 20-30 million people per year worldwide.[1]

## Objectives

The objective of this study was to analyse the burden of septic shock in 2012 for England, Wales and Northern Ireland.

## Methods

We analyzed length of stay, survival and organ support of adult septic shock patient admitted to general critical care units. Septic shock was defined as severe sepsis including the presence of cardiovascular organ dysfunction (from cardiovascular SOFA Score of 2, 3 or 4). Physiological definitions were matched as closely as possible to those used in the PROWESS trial.[2]

These data derive from the Case Mix Programme Database. The Case Mix Programme is the national, comparative audit of patient outcomes from adult critical care coordinated by the Intensive Care National Audit & Research Centre (ICNARC). These analyses are based on data for 136,880 admissions to 205 adult, general critical care units based in NHS hospitals geographically spread across England, Wales and Northern Ireland. For more information on the representativeness and quality of these data, please contact ICNARC.

## Results

Tables 1 and 2.

**Table 1:** Case mix and mortality of admissions.

Number of admissions with septic shock (%)	22,081 (16.1)
Number of patients with septic shock	20,549
Age in years (mean/median)	64.0/67
Gender, percent males	52.6
Total hospital mortality, deaths (%)	7724 (37.6)
- Mortality during first CCU admission, deaths (%)*	5772 (28.1)
- Post first CCU in-hospital mortality, deaths (%)	1952 (9.5)

* Including 1532 re-admissions to CCU, total CCU mortality is 6100 deaths (29.7%).

**Table 2:** Length of hospital stay and organ support in 2012.

Critical care unit average length of stay in days (median) - All	7.6 (4.1)
- Unit survivors	8.2 (4.7)
- Unit non-survivors	5.9 (2.4)
Post-unit average length of stay in days (median) - All	23.3 (31.4)
Average number of organs supported (median)	2.1 (2)
- Percentage receiving continuous renal support	20.1
- Days of renal support (mean/median)	5.4/3
- Percentage receiving advanced respiratory support	62.5
- Days of advanced respiratory support (mean/median)	7.7/4

## Conclusions

It is evident that patients who develop septic shock pose a heavy burden to the UK society, as well as to the NHS hospitals. One in every six patient admitted to a critical care unit is diagnosed with septic shock. Nearly 40 percent ultimately die while staying at the hospital, of which close to 30 percent die during CCU stay. These patients also require substantial renal and respiratory support, as well as total hospital stays of up to a month. Septic shock is a very burdensome and costly illness and every effort should be made to reduce this burden to the patients, hospitals and society.

## Grant Acknowledgment

FA and ALK are employed by Ferring Pharmaceuticals. At the time of the study, TK was employed by Ferring Pharmaceuticals.
